# Image Quality Assessment of Diffusion-Weighted Imaging (DWI) and Its Impact on Apparent Diffusion Coefficient (ADC) as a Quantitative Imaging Biomarker for Predicting Response to Neoadjuvant Chemotherapy in High-Risk Early Breast Cancer

**DOI:** 10.3390/tomography12060087

**Published:** 2026-06-17

**Authors:** Wen Li, Lisa J. Wilmes, Julia Carmona-Bozo, Nu N. Le, Maggie Chung, Jessica E. Gibbs, Natsuko Onishi, Elissa Price, Bonnie N. Joe, John Kornak, Thomas L. Chenevert, Dariya Malyarenko, Patrick J. Bolan, Savannah C. Partridge, Nola M. Hylton

**Affiliations:** 1 Department of Radiology and Biomedical Imaging, University of California, San Francisco, CA 94158, USA; lisa.wilmes@ucsf.edu (L.J.W.); juliauno@hotmail.com (J.C.-B.); nu.le@ucsf.edu (N.N.L.); maggie.chung@ucsf.edu (M.C.); jessica.gibbs@ucsf.edu (J.E.G.); natsuko.onishi@ucsf.edu (N.O.) elissa.price@ucsf.edu (E.P.); bonnie.joe@ucsf.edu (B.N.J.); nola.hylton@ucsf.edu (N.M.H.); 2 Department of Epidemiology and Biostatistics, University of California, San Francisco, CA 94158, USA; john.kornak@ucsf.edu; 3 Department of Radiology, University of Michigan, Ann Arbor, MI 48109, USA; tlchenev@med.umich.edu (T.L.C.);; 4 Department of Radiology, University of Minnesota, Minneapolis, MN 55455, USA; bola0035@umn.edu; 5 Department of Radiology, University of Washington, Seattle, WA 98195, USA; scp3@uw.edu

**Keywords:** breast cancer, diffusion-weighted MRI, apparent diffusion coefficient, image quality, fat suppression, artifact, signal-to-noise ratio, pathologic complete response

## Abstract

Background/Objectives: Apparent diffusion coefficient (ADC) calculated from diffusion-weighted MRI (DWI) can predict tumor response to neoadjuvant chemotherapy for breast cancer. However, obtaining consistently adequate image quality in breast DWI can be challenging, and the effect of image quality on ADC’s predictive performance is unclear. The objective of this study was to evaluate inter-reader variability in image quality assessment and the effect of DWI image quality on the predictive performance of ADC. Methods: This multi-institutional study included 428 patients. Two readers assessed three DWI image quality factors—fat suppression, artifacts, and signal-to-noise ratio (SNR). Inter-reader agreement was estimated using Fleiss’ Kappa. The percent change in tumor ADC from pretreatment (T0) to early treatment (T1) was used to predict pathologic complete response (pCR), assessed at surgery. Results: Out of 428 patients, 134 were excluded (missing pCR [*n* = 17]; missing/incorrect DWI [*n* = 23]; inability to define region-of-interest [ROI, *n* = 94]) and 294 were included in the analysis. Kappa coefficients were estimated as: 0.47 (95% confidence interval [CI]: 0.42, 0.52) for fat suppression, 0.54 (0.50, 0.59) for artifact, and 0.38 (0.32, 0.44) for SNR. The AUC of ADC calculated from DWI with adequate (high or medium at both time points) image quality was 0.61 (95% CI: 0.52, 0.702), while it was 0.68 (95% CI: 0.53, 0.83) from DWI with inadequate image quality at either T0 or T1. The *p*-value for the difference in AUCs was 0.45. Conclusions: The inter-reader agreement was moderate to fair across all three quality categories. When a manually delineated tumor ROI was possible, no statistically significant difference in ADC predictive performance was observed between the quality-adequate and quality-inadequate cohorts; still, both were predictive of pCR. Furthermore, no statistically significant differences were observed in inter-reader agreement or ADC predictive performance between 1.5T and 3T scanners. These findings are clinically relevant to the use of ADC as an imaging biomarker in real-world conditions.

## Simple Summary

Apparent diffusion coefficient (ADC) calculated from diffusion-weighted MRI (DWI) can predict tumor response to neoadjuvant chemotherapy for breast cancer. However, ADC measurements suffer from inadequate image quality. The objective of this study was to evaluate inter-reader variability in image quality assessment and the effect of DWI image quality on the predictive performance of ADC. We analyzed DWI data from a multi-center clinical trial, I-SPY 2. Our results showed fair to moderate inter-reader agreement in the quality ranking for fat suppression, artifacts, and signal-to-noise ratio. When the tumor ROI could be drawn, ADC performance for predicting pathologic complete response did not differ between groups stratified by image quality or MRI scanner field strength.

## 1. Introduction

The apparent diffusion coefficient (ADC), derived from diffusion-weighted MRI (DWI), quantifies microscopic water mobility and reflects tissue cellularity and microstructure [1]. In breast cancer treated with neoadjuvant chemotherapy (NAC), longitudinal changes in tumor ADC relative to baseline ADC have been associated with treatment response [2,3]. In the multicenter ACRIN 6698 study (NCT01564368) conducted within the I-SPY TRIAL (NCT01042379), ADC change midtreatment can predict pathologic complete response (pCR) [4]. Other studies showed that an early (as early as one week after the treatment initiation) increase in mean tumor ADC can predict pCR in breast cancer undergoing NAC [5,6,7,8,9].

Despite this promise, breast DWI is not yet routinely incorporated into clinical assessment of NAC response, in contrast to dynamic contrast-enhanced (DCE) MRI, owing in part to technical challenges and the absence of standardized ADC measurement [10,11]. Breast DWI image quality can be compromised by insufficient fat suppression, tissue heterogeneity with associated susceptibility effects, geometric distortions inherent to single-shot echo-planar imaging, and limited signal-to-noise ratio (SNR) [12,13,14,15]. Suboptimal image quality can bias ADC measurements and introduce uncertainty in the region-of-interest (ROI) delineation, which in turn can potentially degrade ADC in predicting pCR and influence the clinical decision-making based on ADC. The DWI Biomarker Committee of the Quantitative Imaging Biomarker Alliance (QIBA) recently published a profile for using ADC as a quantitative imaging biomarker in clinical applications, emphasizing the need for image quality and protocol adherence [16]. However, the impact of image quality on the performance of tumor ADC in predicting response to NAC remains unknown [17].

In this study, using data from the multicenter I-SPY 2 TRIAL, we conducted a two-step analysis: first, we assessed inter-reader variability in DWI image quality ratings; second, using reader-adjudicated image quality, we evaluated how DWI image quality affected the predictive performance of tumor ADC for assessing treatment response in breast cancer.

## 2. Materials and Methods

This study was performed as part of the I-SPY 2 TRIAL (www.ClinicalTrials.gov registration no. NCT01042370) and followed the Health Insurance Portability and Accountability Act (HIPAA) policy. All patients provided written informed consent prior to screening and a second consent form after randomization to treatment and before treatment was initiated. All participating sites in the multi-institutional I-SPY 2 received approval from their local human study institutional review boards (IRBs) before the start of recruitment.

### 2.1. Study Cohort

**Figure 1:**
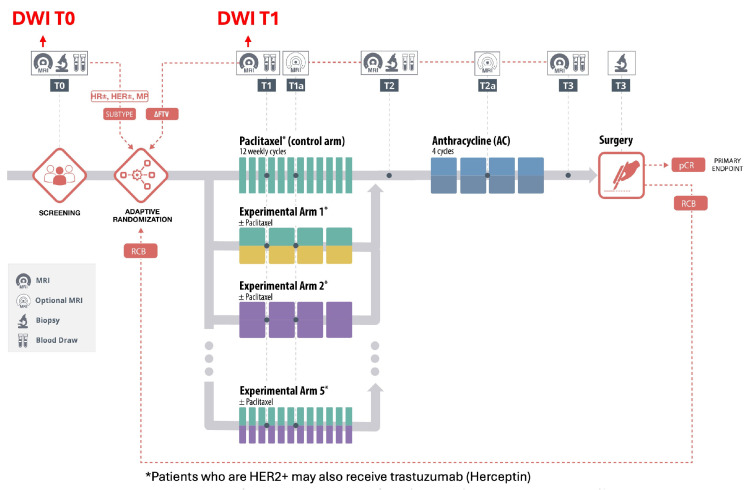
The I-SPY 2 schema and DWI time points in this analysis. Patients enrolled in I-SPY were randomized to one of the treatment arms. After the first regimen, every patient received anthracycline (AC). MRI scans were performed at multiple time points throughout the trial. DW images acquired at T0 (pretreatment) and T1 (early treatment, approximately 3 weeks after the treatment initiation) were evaluated in this study, as marked by red arrows.

This study included a subset of 428 patients from the I-SPY 2 TRIAL. The I-SPY 2 trial is an adaptive, neoadjuvant clinical trial to identify effective new treatments rapidly for high-risk, early-stage breast cancer. Patient eligibility for enrollment is outlined elsewhere [18,19]. In summary, the patient must be at least 18 years old and diagnosed with high-risk (via MammaPrint, Agendia, Irvine, CA, USA), stage II or III invasive breast cancer, featuring tumors ≥2.5 cm. The 428-patient cohort comprised the first four experimental drug arms that graduated from I-SPY 2-Neratinib, Veliparib-Carboplatin, MK-2206, and Pembrolizumab [20,21,22,23]—or their corresponding control arm. See Figure 1 for the I-SPY 2 study schema.

The analysis cohort of this study was determined after data were excluded due to any of the following reasons: (1) Pathological outcome (i.e., pCR status) was missing. (2) DWI was not performed. (3) DWI was not acquired at both b = 0 and 800 s/mm^2^. (4) Region-of-interest (ROI) was not possible due to a lack of confidence in localizing the tumor region.

### 2.2. Pathological Outcome

The pathological outcome was evaluated during surgery, after NAC was completed. See Figure 1 for the surgical time point. A binary outcome, pCR or non-pCR, was used in this study. A pCR was determined by the absence of residual invasive cancer in both the breast and lymph nodes, as confirmed through pathological examination of the surgical specimen.

### 2.3. DWI Acquisition and Analysis

MRI exams were performed at multiple treatment time points in I-SPY 2. They were pretreatment (T0), early treatment (T1, 3 weeks), inter-regimen (T2, 12 weeks), and pre-surgery (T3). According to the I-SPY 2 imaging protocol, a 1.5 or 3.0 Tesla scanner with a dedicated breast radiofrequency coil was allowed to acquire MR images, and all MRI exams of a given patient were required to be performed on the same scanner configuration (model, field strength, and breast coil model) [24]. Patients were imaged in the prone position. The image acquisition protocol included a localization scan and three acquisitions: T2-weighted, DWI, and DCE-MRI. Only DWI exams at T0 and T1, in which tumor sizes were relatively larger than in later time points (T2 or T3), were evaluated in this study because larger tumors could help us avoid the partial-volume effect when we measured tumor ADC. See Figure 1 for the I-SPY 2 schema and DWI time points analyzed. Each patient went through screening, adaptive randomization, first regimen (paclitaxel only or paclitaxel ± experimental drugs), second regimen (AC), and surgery.

Details of DWI acquisition can be found in Table S1. In summary, a bilateral and fat-suppressed single-shot EPI sequence with parallel imaging (acceleration factor = 2) was used. The recommended field of view was 260 to 360 mm, with an acquired matrix of 128 × 128 to 192 × 192. The recommended acquired in-plane resolution was 1.9 mm or higher, and slice thickness was 3–5 mm. The total imaging time was 5 min or less. Diffusion gradients applying at least two b-values: b = 0 and b = 800 s/mm^2^ were required. DWI analysis was performed at the imaging core lab using in-house software written in the IDL programming language (Exelis Visual Information Solutions, Boulder, CO, USA), in which ADC maps were calculated using DWI images acquired with b = 0 and b = 800 s/mm^2^ images: $ADC=-\frac{1}{b}ln\frac{S_{b}}{S_{0}}$, where b = 800 and $S_{b}$ represents signal intensity at b = 800, and $S_{0}$ represents signal intensity at b = 0.

Breast tumors were identified using the subtraction image calculated from the pre- and first post-contrast DCE images. Tumor ROIs were delineated on ADC maps using the multi-slice restricted ROI technique reported by Le et al. [25]. Specifically, the tumor ROI was defined manually by encompassing the most diffusion-restricted area indicated by DWI on all applicable slices (i.e., dark on ADC map and bright on b = 800 s/mm^2^ diffusion-weighted image). In addition, special care was taken to avoid cystic, necrotic, or fatty components by excluding areas with no contrast enhancement on DCE-MRI. Tumor ROIs were re-delineated at T1 using T0 ROIs as a reference. Tumor ADC was calculated as the average of ADC values over the entire tumor ROI volume. The ADC change was calculated as $\frac{Tumor\_ADC\_T1\;-\;Tumor\_ADC\_T0}{Tumor\_ADC\_T0}\times 100\%$, where Tumor_ADC_T0 and Tumor_ADC_T1 represent tumor ADC from T0 and T1, respectively.

### 2.4. Image Quality Assessment

The image quality of DWI in the tumor area was visually evaluated and ranked by two readers with 11 and 7 years of experience in analyzing DW images, respectively. Both readers were blinded to tumor clinical characteristics, patient characteristics, and pathologic outcomes. The quality evaluation focused on three categories that often have issues in clinical breast DWI [11]: (1) quality of fat suppression, (2) severity of artifacts (including but not limited to clip artifacts, distortion, ghosting, and displacement between b images), and (3) signal-to-noise ratio (SNR). Fat suppression and artifacts were assessed using both b = 0 and b = 800 images, while the SNR was evaluated using b = 0 images only. A score of 1, 2, or 3 was assigned to each category, representing low, medium, or high image quality, respectively. Details of the quality assessment and example images for each category are provided in Figure S1 and Table S2, which were also used as training materials for image quality assessment. Both readers assessed the image quality independently, and skip-level discrepancies (1–3 or 3–1) were resolved by reviewing images and discussing them in person. A third reader, a radiologist specializing in breast imaging, was recruited to assess the image quality of a small cohort (a randomly selected subset of this study cohort) for additional analysis of the inter-reader variability. The third reader was trained using the same materials as Readers 1 and 2. However, skip-level discrepancies were not addressed using the approach described above because the third reader’s assessment was not incorporated into the analysis of the effect of image quality on the predictive performance of tumor ADC. Please see the details in Figure S1.

**Figure 2:**
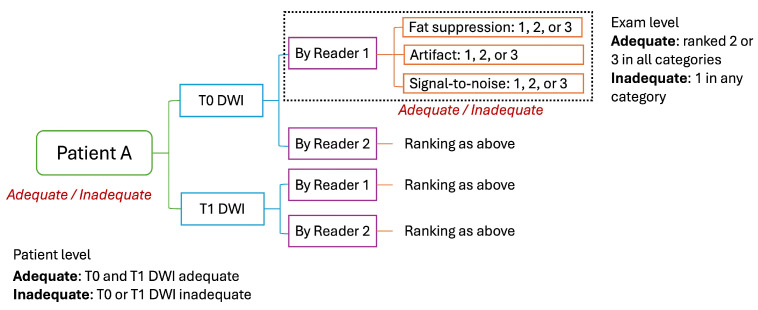
Quality categories at the exam level and at the patient level. For each patient, DWI exams acquired at T0 (pretreatment) and T1 (early treatment) were ranked by two readers (Reader 1 and Reader 2). Each reader ranked independently in 3 categories (fat suppression, artifacts, signal-to-noise ratio). The exam-level binary ranking (adequate/inadequate) was defined using rankings in all 3 categories for each reader (dotted box). T0 DWI would be ranked inadequate by Reader 1 if any category had a “1” and this exam would be ranked adequate by Reader 1 otherwise. The patient-level binary ranking (adequate/inadequate) was defined by the ranking of T0 and T1 DWI. The patient was categorized into the inadequate cohort if T0 or T1 DWI was ranked inadequate (by both readers).

At the exam level, a binary ranking was generated by consolidating the three-level ranking by each reader: (1) a DWI exam ranked as “1” in any category was classified as having “inadequate” image quality; (2) a DWI exam ranked as “2” or “3” in all categories was classified as having “adequate” image quality (Figure 2). The purpose of using binary ranking at the exam level was to assess inter-reader agreement on eliminating exams from the ADC analysis due to inadequate image quality.

At the patient level, the analysis cohort was divided into Inadequate and Adequate cohorts: the Inadequate cohort included patients with DWI exams ranked “inadequate” by both readers at T0 or T1, and the Adequate cohort included the remaining patients, who could have DWI exam(s) ranked “inadequate” by only one reader. The purpose of the patient-level binary ranking was to examine the effect of poor image quality, confirmed by both readers, on the predictive performance of ADC for treatment response, with ADC changes from T0 to T1 analyzed. The Inadequate cohort was further divided into two sub-cohorts: Inadequate sub1 and Inadequate sub2, where sub1 included patients with inadequate DWI quality at both T0 and T1, and sub2 included patients with inadequate DWI quality at either T0 or T1, but not both.

Due to the subjectiveness of the qualitative ranking for image quality, we compared ADC performance in two Consensus groups—Consensus Adequate, including patients with exams ranked adequate by both readers at both T0 and T1, and Consensus Inadequate, including patients with exams ranked inadequate by both readers at both T0 and T1, equivalent to the Inadequate sub1 defined above.

### 2.5. Statistical Analysis

For patient characteristics, age was summarized as mean ± standard deviation (SD) and a two-sided Student *t*-test compared mean age differences between two groups. Categorical patient characteristics (cancer subtype, treatment, menopausal status, pCR status) were compared using a two-sided Fisher’s test.

The inter-reader agreement was estimated using Cohen’s Kappa, with Fleiss–Cohen weights. Cohen’s Kappa was used as a statistical measure for categorical rankings (i.e., fat suppression, artifacts, SNR) between two readers. It has values ranging from −1 to 1, where 1 indicates perfect agreement and 0 indicates agreement equivalent to random chance. When the Kappa value is negative, it indicates that observed agreement between readers is worse than that expected by chance. Implementation of Fleiss–Cohen weights allowed less penalty to be placed on disagreements that were not far apart. Percent agreement was calculated for binary rankings (adequate versus inadequate).

Due to the non-Gaussian distribution of ADC changes observed, we reported them using the median and interquartile range. The Wilcoxon rank-sum test was used to estimate differences in ADC changes in pCR versus non-pCR patients. The association between ADC change and pCR was evaluated by the area under the receiver operating characteristic curve (AUC), where we assumed that patients who achieved pCRs had higher ADC change than patients who had non-pCRs.

To evaluate whether the association between ADC change and pCR differed by biological subtype, we performed multivariable logistic regression analyses including breast cancer subtype and an interaction term between subtype and ADC change, in which the breast cancer subtype was defined by hormone receptor (HR) and human epidermal growth factor receptor 2 (HER2) statuses: HR+/HER2−, HER2+, triple-negative breast cancer (TNBC). The interaction term in a logistic regression model was evaluated by fitting two models, one with and one without the interaction, and comparing their fits using the likelihood ratio test.

All statistical analyses were conducted in R (version 4.5.1, http://www.r-project.org). A *p*-value less than 0.05 was considered a statistically significant result.

## 3. Results

### 3.1. Patient Characteristics

**Figure 3:**
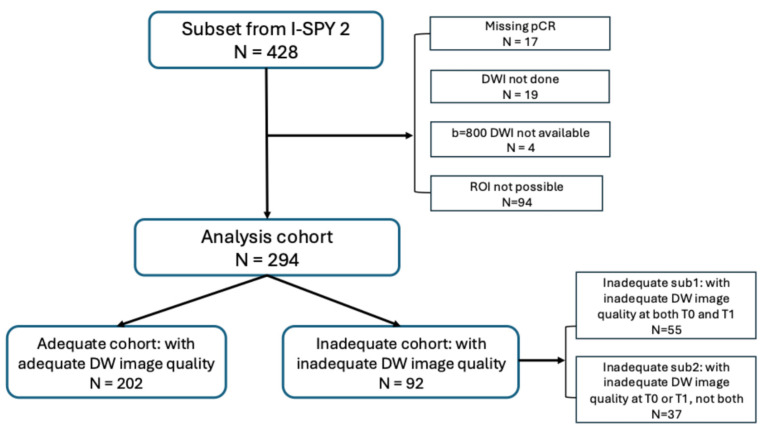
Study cohort inclusion-and-exclusion flowchart.

The final analysis cohort included 294 patients after 134 (31%) were excluded from the 428 total from the I-SPY 2 trial—*n* = 17 missing pCR, *n* = 19 missing DWI exams, *n* = 4 missing b = 800 DWI, and *n* = 94 missing tumor ROI. See Figure 3 for the data inclusion-and-exclusion flowchart. Table 1 shows patient characteristics for the analysis cohort (*n* = 294) and the excluded cohort (*n* = 134), who were excluded from the 428-patient whole cohort. In summary, age, HER2 status, and menopausal status were not statistically different between the two cohorts. However, HR status, treatment arms, and pCR status differed significantly.

### 3.2. Inter-Reader Agreement

**Figure 4:**
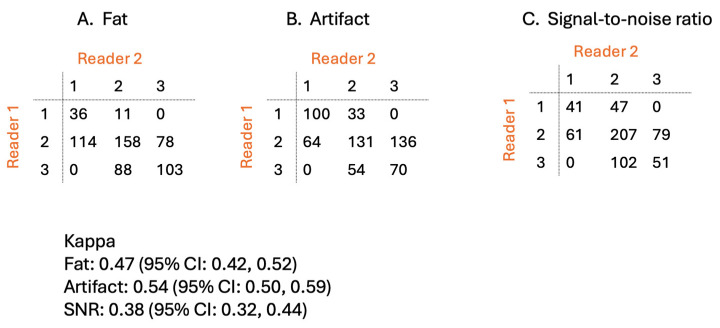
Inter-reader agreement for DWI image quality. Each reader ranked 588 diffusion-weighted MRI exams acquired at pretreatment and early-treatment time points for 294 patients, with two exams per patient. A score of 1, 2, or 3 was given to rank the image quality of fat suppression (**A**), artifacts (**B**), and signal-to-noise (SNR) (**C**): “1” for low, “2” for medium, and “3” for high image quality. The numbers on the main diagonal of the matrix counted the number of agreements, and the off-diagonal numbers counted the number of disagreements.

Two readers independently reviewed the DW images and ranked the image quality of 588 DWI exams (two exams per patient). See Figure 4 for the agreement between the two readers and Figure S2 for the agreement between two out of three readers. See Figures S3–S5 for the inter-reader agreement by field strength, age, and chemotherapy regimen.

For fat suppression (Figure 4A), Cohen’s Kappa was 0.47 (95% confidence interval [CI]: 0.42, 0.52) and the percent agreement was 50.5% (297/588) in the three-level ranking. The percent agreement was 79% when the three-level ranking was consolidated to two levels: inadequate versus adequate fat suppression. Reader 1 considered 47 exams (8%) to have inadequate fat suppression (ranked 1), and Reader 2 considered 150 exams (26%) to have inadequate fat suppression. Reader 1 was more conservative compared to Reader 2 (*p* < 0.001). Both readers agreed that 36 exams (6%) had inadequate fat suppression.

For artifacts (Figure 4B), Cohen’s Kappa was 0.54 (95% CI: 0.50, 0.59) and the percent agreement was also 51.2% (301/588) in the three-level ranking. Reader 1 considered 133 exams (23%) to have inadequate quality (ranked 1 for artifacts), and Reader 2 considered 164 exams (26%) to have inadequate quality. Reader 1 was more conservative compared to Reader 2 (*p* = 0.044). Both readers agreed that 100 exams (17%) had inadequate quality because of artifacts.

For SNR (Figure 4C), Cohen’s Kappa was 0.38 (95% CI: 0.32, 0.44) and the percent agreement between the two readers was 50.9% (299/588) in the three-level ranking. Reader 1 considered 88 exams (15%) to have inadequate SNR (ranked 1), and Reader 2 considered 102 exams (17%) to have inadequate SNR. There was no statistically significant difference regarding the proportion of inadequate SNR exams ranked by two readers (*p* = 0.303). Both readers agreed that 41 exams (7%) had inadequate SNR.

**Figure 5:**
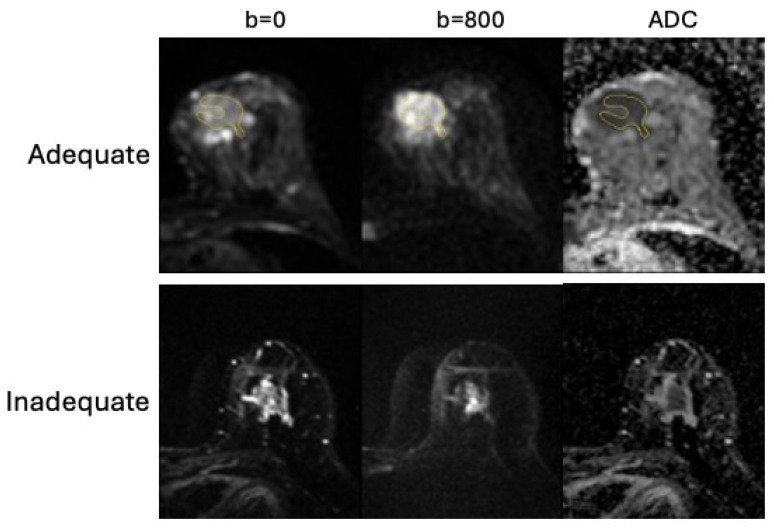
Example cases of diffusion-weighted MRI exams with adequate and inadequate image quality. A representative slice with the largest tumor area is selected for each case, and the images for the adequate case are overlaid with a manually delineated region of interest in yellow. The adequate case was classified as being of adequate quality by both readers, and the inadequate case was classified as being of inadequate quality by both readers.

At the exam level, the percent agreement between two readers was 79% for the binary ranking (adequate versus inadequate). Reader 1 considered 197 exams (34%) to have inadequate image quality, and Reader 2 considered 223 exams (38%) to have inadequate image quality. The difference between Reader 1 and Reader 2 was not statistically significant (*p* = 0.13). Both readers agreed that 147 exams (25%) had inadequate image quality. Table S3 shows the distribution of field strength across these 147 exams. Example cases of adequate and inadequate exams are shown in Figure 5.

### 3.3. Predictive Performance of ADC

Out of 294 patients (Full cohort), 92 (31%) were classified as having inadequate DW image quality (referred to as the Inadequate cohort), while the remaining 202 (69%) patients were classified as having adequate image quality (referred to as the Adequate cohort). In the Full cohort, the pCR rate was 29% (86/294), whereas it varied slightly in the Adequate cohort (31%, 62/202) and the Inadequate cohort (26%, 24/92), with no difference between the cohorts (*p* = 0.49). Comparisons of other patient characteristics between the Adequate and Inadequate cohorts are shown in Table 2. The mean age in the Adequate cohort was 47 ± 11 (SD), while it was older (51 ± 11) in the Inadequate cohort, *p* = 0.003. The number of patients with HER2+ breast cancer was four (2%) in the Adequate cohort, while it was eight (9%) in the Inadequate cohort, *p* = 0.011. However, the numbers were relatively small compared to HER2−.

**Figure 6:**
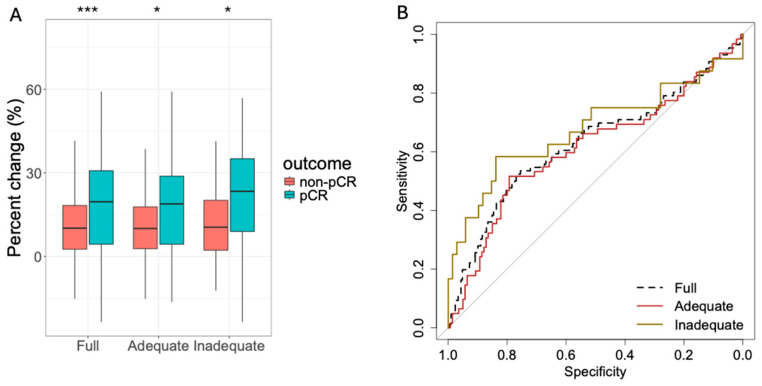
Predicting pathologic complete response (pCR) by ADC change. (**A**) shows box plots of percent change in ADC in pCR versus non-pCR groups extracted from the entire analysis cohort (Full), adequate-image-quality sub-cohort (Adequate), and inadequate-image-quality sub-cohort (Inadequate). *p*-values were <0.001 (***), 0.012 (*), and 0.0103 (*) for the difference between pCRs and non-pCRs, respectively. The statistically significant levels are labeled at the top. (**B**) shows ROC curves of percent change in ADC in predicting pCR in the corresponding cohorts. The area under the ROC curve (AUC) was 0.63 (95% confidence interval [CI]: 0.55, 0.704), 0.61 (95% CI: 0.52, 0.702), and 0.68 (95% CI: 0.53, 0.83), respectively.

Associations between ADC change and pCR status in the Full cohort and in the Adequate/Inadequate cohorts are shown in Table 3. All three cohorts showed larger ADC changes in the pCR groups than in the non-pCR groups (Figure 6A), and the differences were all statistically significant: 7.4% (95% CI: 3.1%, 11.6%, *p* < 0.001) in the Full cohort, 6.04% (95% CI: 1.4%, 10.8%, *p* = 0.012) in the Adequate cohort, and 11.4% (95% CI: 2.8%, 19.8%) in the Inadequate cohort. Estimated AUCs were comparable and all statistically significantly above 0.5, ranging from 0.61 to 0.68. The AUC in the Inadequate cohort was estimated to be 0.68 (95% CI: 0.53, 0.83), higher than the estimated AUC in the Adequate cohort, AUC = 0.61 (95% CI: 0.52, 0.702). However, the difference was not statistically significant (*p* = 0.45). Figure 6B shows the corresponding ROCs. Table S4 shows AUCs for predicting pCR using ADC change by field strength, age, and chemotherapy regimen.

The Inadequate cohort was further divided into two image quality sub-cohorts, and the association between ADC change and pCR in sub-cohorts can be found in Table S5 and Figure S6). In the Inadequate sub2 cohort (inadequate quality at either T0 or T1, not both), statistically significant difference in ADC change between pCR and non-pCR groups was observed: 16.4% (95% CI: 5.9%, 26.1%, *p* = 0.0055); meanwhile, the difference was not statistically significant in the Inadequate sub1 cohort (inadequate quality at both T0 or T1): 3.7% (95% CI: −7.5%, 19.4%, *p* = 0.45). AUCs were 0.57 (95% CI: 0.35, 0.80) in Inadequate sub1 and 0.78 (95% CI: 0.60, 0.96) in Inadequate sub2, *p* = 0.16 for the difference in AUCs. When the Inadequate sub1 was combined with the Adequate cohort (*n* = 239 in the combined cohort), the AUC of predicting pCR was 0.64 (95% CI: 0.56, 0.72; *p* < 0.001) for ADC change. Table S5 also shows the association between ADC change and pCR in the Consensus Adequate group, in which the AUC was 0.60 (95% CI: 0.49, 0.71), while the AUC estimated in the Consensus Inadequate (equivalent to Inadequate sub1) was 0.57 (95% CI: 0.35, 0.80), *p* = 0.83 for the difference in AUCs.

Likelihood ratio tests showed that the interaction between ADC change and breast cancer subtype was statistically significant in the Inadequate cohort (*p* = 0.011), while it was not statistically significant in the Adequate cohort (*p* = 0.95), suggesting that the relationship between ADC change and pCR may vary across molecular subtypes rather than be fully attributable to image quality alone. See Table S6 for the subtype distribution in the Adequate and Inadequate cohorts, and Figure S7 for the R code and detailed results.

## 4. Discussion

Our reader study found moderate to fair agreement between the two readers regarding fat suppression and artifacts, but lower agreement for SNR. No difference in inter-reader agreement was found in images acquired by 1.5T and 3T scanners. Furthermore, no statistically significant difference was found in predicting pCR when ADC changes were derived from quality-adequate or quality-inadequate DWI images. ADC was predictive in both groups.

The image quality assessment developed in this study focused on three categories: fat suppression, artifacts, and SNR. These are known issues in clinical breast DWI. A secondary analysis using the data from the ECOG-ACRIN Cancer Research Group Multisite Trial (A6702) reported factors affecting image quality and lesion evaluability in breast DWI [11]. Multiple image quality factors were considered: fat suppression, SNR, image artifacts, and misregistration within the DWI series (categorized as an artifact in our study). Quality issues related to these factors were found in 11% to 24% of the scans they reviewed (103 in total). In the ACRIN 6698 trial, all DWI images were assessed based on three quality categories: artifacts, fat suppression, and SNR. Images with poor quality in any of these categories were excluded from the primary analysis [26].

Our results found moderate to poor agreement between two readers in the three-level (1—low; 2—medium; 3—high) quality assessment of the individual categories of fat suppression, artifacts, and SNR, which was confirmed by the additional reader (see Figure S2). This finding suggested that qualitative assessments were subjective. Without understanding the fundamental issue causing the inadequate quality, a reader may find it difficult to differentiate inadequate fat suppression from an artifact because poor fat suppression can cause chemical shift artifacts or image distortion [27]. The agreement increased when the quality ranking was used to eliminate low-quality exams, reaching 79%. Most published studies used an overall ranking with levels 1 to 5 for breast DWI in assessing the inter-reader agreement. Bickel et al. reported an intraclass correlation (ICC) of 0.50–0.67 between any two of four readers when they used a score of 1–5 to visually rank the breast DWI image quality [28]. It was a single-institution, single-scanner study. However, another single-institution, single-scanner study conducted by Ahn et al. evaluated the image quality of breast DWI using a comprehensive multi-factor, multi-level method [29]. Between the two readers, their study found the lowest agreement in evaluating background noise, the highest in evaluating ghosting artifacts, and fat suppression in between, consistent with our study.

Among the three image-quality categories, SNR had the lowest inter-reader agreement (i.e., the lowest Cohen kappa), and artifacts showed the highest agreement. The low Cohen Kappa indicates that qualitative visual assessment is unreliable for identifying SNR limitations in breast DWI and underscores the need for objective metrics. The European Society of Breast Radiology (EUSOBI) International Breast DWI working group demonstrated that low SNR can lead to underestimated ADC values due to Rician bias [30]. Accordingly, a sufficiently high SNR is necessary for accurate ADC measurement and for utilizing ADC to assess treatment response. However, accurately quantifying SNR in a clinical trial remains challenging. The impact of artifacts on ADC depends on the type of artifact. For example, the displacement between images acquired at different b-values (e.g., due to eddy currents) can cause spatial misregistration and alter ADC values at tissue boundaries, either increasing or decreasing them locally. Fat suppression had the second-highest level of disagreement based on the Kappa values in this study. Fat suppression is a unique challenge for breast MRI, due to the high proportion of fat in breasts. Insufficient fat suppression will contaminate the water diffusion signal because fat has a low apparent diffusion coefficient, residual fat signal biases the diffusion signal decay and it can artifactually lower ADC values [27]. In this study, visual assessment of fat suppression was performed by referencing DCE-MRI T1-weighted images to identify fat and glandular regions, then evaluating whether fat was sufficiently suppressed on the b = 0 and b = 800 s/mm^2^ images based on the expected contrast between fat and parenchyma.

In contrast to a reader study, a phantom study can provide objective quality assessments for breast DWI, especially for multicenter clinical trials. The ice-water DWI phantom used for absolute ADC accuracy assessment and scanner qualification in the 6698 study [4] did not mimic the breast tissue complexity needed for assessment of artifacts and fat suppression. A dedicated breast phantom can mimic the geometry and properties of human breast tissue, lesions, and fat with known ADC values [31]. Ideally, the phantom can quantify fat suppression, SNR, image distortion, and the presence of other artifacts such as ghosting. In future studies, we will investigate the correlation between qualitative performance assessment by readers and quantitative assessment by breast phantom, and implement regular phantom scans to identify and address quality issues in the I-SPY 2 trial.

An important application of the quality ranking system is to eliminate low-quality imaging data from the imaging biomarker analysis so that the biased measurements are not included. In this study, the effect of low-quality imaging data on the performance of ADC changes for predicting treatment response in breast cancer NAT was observed only when both time points used to calculate the ADC change had low-quality DWI (Inadequate sub1). The effect was not observed in the Inadequate sub2 cohort, in which low quality occurred at only one time point. In addition, ADC derived from the highest-image-quality DWI (Consensus Adequate) was not found to be more predictive of pCR than ADC derived from the lowest-image-quality DWI (Consensus Inadequate), *p* = 0.83. This result suggested that ADC change as an imaging biomarker for predicting treatment outcomes was robust and resilient against low image qualities if tumor ROIs could be confidently delineated. In this study, tumor ROIs were manually delineated by experienced breast imaging experts, thereby compensating for image quality issues by avoiding ambiguous (questionable) voxels [25].

Interestingly, the highest and the lowest numeric AUCs (0.78 and 0.57) were observed in the two low-quality groups (Inadequate sub2 and sub1, respectively). However, the difference between them was not statistically significant (*p* = 0.16). Considering the small sample sizes (12 pCRs in both Inadequate sub1 and sub2), no definitive conclusions can be drawn about the difference in ADC performance in these groups, except that a wide range of ADC changes was observed, which indicated the variability of ADC values measured in low-quality DWIs.

The multivariate analysis in our study showed a potential interaction between ADC change and molecular breast cancer subtype in the image-quality-inadequate cohort, suggesting the role of molecular subtype in addition to image quality in the prediction of pCR using ADC change in this cohort. However, because of the relatively small sample size, this analysis should be considered exploratory. A full multivariate analysis of interactions among ADC change, molecular subtype, and image quality with a larger sample size is warranted for a future study.

Our study did not find any statistically significant differences in the inter-reader agreement or ADC predictive performance when the analysis cohort was stratified by field strength. Nor did we find any difference in the distribution of Adequate and Inadequate exams by field strength. This finding is consistent with other published studies [11,32]. This result suggested that ADC was a stable imaging biomarker across field strengths and could be used reliably across diverse clinical sites, not just at academic centers with high-field scanners, which is essential for many communities that have no access to high-field scanners or for patients with implants, SAR limits, or claustrophobia to be scanned on lower field strengths without sacrificing accuracy.

The results of this multicenter study suggested that 149 patients (94 with ROI not possible and 55 with inadequate image quality, even with ROI possible) were excluded, resulting in 22% data loss due to image quality when ADC was used as a biomarker for assessing treatment response in breast cancer. The data loss rate reported here severely limits the clinical utility and generalizability of ADC, underscoring the need for real-time quality assurance and quality control (QA/QC) in multicenter trials involving imaging biomarkers.

This study has several limitations:

First, even with a standardized imaging protocol in place, DWI exams lacked quality control and real-time quality assessment within the I-SPY 2 trial. The ice-water diffusion phantom used for initial system qualification did not allow evaluation of fat suppression and artifacts. Dedicated breast phantoms were distributed to a subset of I-SPY sites for regular quality assessment, but the data used in this study were all collected before the phantom was distributed. 

Second, potential variability unrelated to image quality could also contribute to the differences observed in the Adequate and Inadequate cohorts. This variability could stem from, but was not limited to, variations in imaging protocols implemented at sites and the use of various scanners from different vendors. 

Third, MRI exams were acquired at four treatment time points in I-SPY 2. However, we only analyzed the first two time points—pretreatment and early treatment. This is a limitation because the effect of DWI image quality on ADC prediction of pCR at later treatment was not evaluated. 

Fourth, a relatively large cohort (*n* = 94) was excluded due to the tumor ROI being impossible, which suggests bias, such as differences in HR status and treatment arms distributions between the analysis cohort and the excluded cohort. 

Fifth, we evaluated the effect of image quality on the most commonly used DWI-based biomarker, mean tumor ADC. Other biomarkers, such as ADC histogram, diffusion coefficient (D), pseudodiffusion coefficient (D*), and perfusion fraction (f) derived from the intravoxel incoherent motion (IVIM) model, will be examined in future studies.

## 5. Conclusions

If DWI exams were analyzable with a manually delineated tumor ROI, the inter-reader agreement was moderate to fair across all three quality categories. No statistically significant difference in predictive performance of ADC was found between the quality-adequate and quality-inadequate cohorts; still, both were predictive of pCR. In addition, we did not observe statistically significant differences in inter-reader agreement or ADC predictive performance between 1.5T and 3T scanners. These findings are clinically important for practice under real-world conditions, where imperfect image quality or lower-field-strength scanners may be used to assess treatment response.

## Figures and Tables

**Table 1:** Patient characteristics in the analysis and excluded cohorts.

Characteristic	Analysis Cohort (*n* = 294)	Excluded Cohort (*n* = 134)	*p* ^1^
Age (mean ± standard deviation)	48 ± 11	49 ± 10	0.32
HR status			0.016
HR+	156 (53%)	54 (40%)	
HR−	138 (47%)	80 (60%)	
HER2 status			0.62
HER2+	12 (4%)	7 (5%)	
HER2−	282 (96%)	127 (95%)	
Treatment			<0.001
Experimental arm	148 (50%)	95 (71%)	
Standard arm	146 (50%)	39 (29%)	
Menopausal status			0.98
Premenopausal	149 (51%)	69 (51%)	
Postmenopausal	95 (32%)	44 (33%)	
Perimenopausal	12 (4%)	4 (3%)	
Others	38 (13%)	17 (13%)	
Pathological outcome			<0.001
pCR	86 (29%)	42 (31%)	
non-pCR	208 (71%)	75 (56%)	
Unknown	0 (0%)	17 (13%)	

^1^ *p*-value was calculated using a two-sided *t*-test for age and a two-sided Fisher test for all other patient characteristics. HR: Hormone receptor. HER2: Human epidermal growth factor receptor 2. pCR: Pathologic complete response. Adequate Cohort: Patients with adequate diffusion-weighted image quality based on two readers’ rankings. Inadequate Cohort: Patients with inadequate diffusion-weighted image quality (poor quality) based on the two readers’ rankings.

**Table 2:** Patient characteristics in the Adequate and Inadequate cohorts.

Characteristic	Adequate Cohort (*n* = 202)	Inadequate Cohort (*n* = 92)	*p* ^1^
Age (mean ± standard deviation)	47 ± 11	51 ± 11	0.003
HR status			0.90
HR+	108 (53%)	48 (52%)	
HR−	94 (47%)	44 (48%)	
HER2 status			0.011
HER2+	4 (2%)	8 (9%)	
HER2−	198 (98%)	84 (91%)	
Treatment			0.62
Experimental arm	104 (51%)	44 (48%)	
Standard arm	98 (49%)	48 (52%)	
Menopausal status			0.16
Premenopausal	111 (55%)	38 (41%)	
Postmenopausal	60 (30%)	44 (33%)	
Perimenopausal	8 (4%)	4 (3%)	
Others	23 (11%)	17 (13%)	
Pathological outcome			0.49
pCR	62 (31%)	24 (26%)	
non-pCR	140 (69%)	68 (74%)	

^1^ *p*-value was calculated using a two-sided *t*-test for age and a two-sided Fisher test for all other patient characteristics. HR: Hormone receptor. HER2: Human epidermal growth factor receptor 2. pCR: Pathologic complete response. Adequate Cohort: Patients with DWI exams ranked “adequate” by at least one reader at both T0 and T1. Inadequate Cohort: Patients with DWI exams ranked “inadequate” by both readers at T0 or T1.

**Table 3:** Association between ADC change and pCR in image quality cohorts.

Cohort	pCR		Non-pCR		Diff of ADC Change (95% CI) (%)	AUC (95% CI)	*p*
	*n*	ADC Change (Median [IQR]) (%)	*n*	ADC Change (Median [IQR]) (%)			
Full	86	19.6 (4.4, 30.7)	208	10.2 (2.6, 18.3)	7.4 (3.1, 11.6)	0.63 (0.55, 0.704)	<0.001
Adequate	62	18.9 (4.4, 28.8)	140	10.04 (2.8, 17.8)	6.04 (1.4, 10.8)	0.61 (0.52, 0.702)	0.012
Inadequate	24	23.3 (8.99, 35.05)	68	10.5 (2.3, 20.1)	11.4 (2.8, 19.8)	0.68 (0.53, 0.83)	0.0103

ADC: Apparent diffusion coefficient. pCR: Pathologic complete response. *n*: Number of patients. Full: The entire analysis cohort of 294 patients. Adequate cohort: Patients with adequate diffusion-weighted image quality at both T0 and T1 based on two readers’ rankings. Inadequate cohort: Patients with inadequate diffusion-weighted image quality (poor quality) at either T0 or T1 based on two readers’ rankings. AUC: Area under the ROC curve. CI: Confidence interval. Diff: Median differences between pCR and non-pCR groups, with estimated 95% confidence intervals. IQR: Interquartile range.

## Data Availability

Part of the original imaging data presented in this study are openly available in the Cancer Imaging Archive (TCIA) at https://www.10.7937/TCIA.D8Z0-9T85 and https://www.10.7937/tcia.kk02-6d95. We are currently sharing the remaining data publicly on TCIA. Before that, the data is available upon request from the corresponding author.

## Supplementary Materials

The following supporting information can be downloaded at https://www.mdpi.com/article/10.3390/tomography12060087/s1: Figure S1: Example images with different quality rankings; Figure S2: Inter-reader agreement of DW image quality among 3 readers; Figure S3: Inter-reader agreement for DW image quality by field strength; Figure S4: Inter-reader agreement for DW image quality by age group; Figure S5: Inter-reader agreement for DW image quality by chemotherapy regimen; Figure S6: Boxplots of ADC change versus pathologic complete responses in the quality inadequate sub-cohorts; Figure S7: Multivariate analysis of interaction between ADC change and breast cancer molecular subtype (HR+/HER2−, HER2+, TNBC); Table S1: Standardized I-SPY 2 DWI acquisition parameters; Table S2: Diffusion-weighted image quality ranking description; Table S3: MRI scanner field strength used to acquire data for Adequate and Inadequate exams; Table S4: AUCs of predicting pCR using ADC change by field strength, patients’ age, and chemotherapy regimen; Table S5: Association between ADC change and pCR in image quality inadequate sub-cohorts; Table S6: Subtype distributions across quality groups.
