# Supplementary material for: Image Quality Assessment of Diffusion-Weighted Imaging (DWI) and Its Impact on Apparent Diffusion Coefficient (ADC) as a Quantitative Imaging Biomarker for Predicting Response to Neoadjuvant Chemotherapy in High-Risk Early Breast Cancer
Source: Tomography. 2026 Jun 17;12(6):87. doi: 10.3390/tomography12060087 (PMC13306497; doi:10.3390/tomography12060087)
Supplement: Supplementary file 1 [file tomography-12-00087-s001.zip › tomography-4167578-supplementary.pdf]

Table S1 Standardized I-SPY 2 DWI acquisition parameters

	**DWI**
Pre or post DCE	Pre DCE
Sequence type	Diffusion-weighted spin echo, echo planar imaging (DW SE-EPI)
2D or 3D sequence	2D
Slice orientation	Axial
Laterality	Bilateral
Frequency direction	R/L
Phase direction	A/P
FOV - frequency	260 – 360 mm
FOV - phase	260 – 360 mm
Matrix – frequency (acquired)	128 – 192
In-plane resolution	≤ 1.9 mm
Fat-suppression	Active fat-sat
TR	≥ 4,000 ms
TE	Minimum TE (50 – 100 ms)
Flip angle	90 degrees
B values	0, 800 s/mm^2
Slice thickness (acquired, not interpolated)	3 – 5 mm
Number of slices	Variable; complete bilateral coverage
Slice Gap	≤ 1.0 mm
Parallel imaging factor	≥ 2
No. of excitations or averages	≥ 2 (to achieve approx. 4 min scan duration)

Table S2 Diffusion-weighted image quality ranking description.

Quality score	Fat suppression<br>*Assessed on b=0 and b=800 images, using T1 or T2 as a reference to identify fat tissue*	Artifact<br>*Assessed on b=0 and b=800 images. Included but not limited to clip artifacts, distortion, ghosting, displacement (between b images)*	Signal-to-noise ratio (SNR)<br>*Assessed tumor and tissue contrast to background on b=0 image only, using T1 or T2 as a reference.*
1	Fat suppression failed or severe enough that tumor ADC can't be trusted	Severe artifacts in tumor area. Tumor ADC can't be trusted.	Very poor SNR at tumor area. Tumor ADC can't be trusted.
2	Fat was partially suppressed	Artifact can be seen near tumor	Noisy in tumor area
3	No obvious fat seen in the image	No artifact seen anywhere near the tumor	Good SNR

The image quality of each DWI study was rated in each of the 3 categories: fat suppression, artifact, and signal-to-noise ratio (SNR). A score of 1, 2, or 3 was given to each category: 1 poor; 2 acceptable; 3 good. T1: T1-weighted MRI; T2: T2-weighted MRI. ADC: apparent diffusion coefficient.

Table S3 MRI scanner field strength used to acquire data for Adequate and Inadequate exams. Fisher's test showed no statistically significant association between image quality (Adequate / Inadequate) and field strength (1.5 T / 3.0 T), p = 0.12.

Field Strength (Tesla)	Adequate	Inadequate
1.5	346 (78%)	106 (72%)
3.0	95 (22%)	41 (28%)

Table S4 AUCs of predicting pCR using ADC change by field strength, patients' age, and chemotherapy regimen

	N	pCR rate	AUC (95% CI)	P
Analysis cohort	294	29% (86/294)	0.63 (0.55, 0.704)	
By field strength				0.29
1.5T	226	28% (63/226)	0.61 (0.52, 0.70)	
3.0T	68	34% (23/68)	0.70 (0.55, 0.85)	
By patients' age				0.37
< 50 years old	150	27% (40/150)	0.59 (0.47, 0.71)	
>= 50 years old	141	32% (45/141)	0.66 (0.56, 0.77)	
By chemotherapy regimen				0.60
Experimental	148	38% (56/148)	0.60 (0.50. 0.70)	
Standard	146	21% (30/146)	0.64 (0.51, 0.77)	

Table S5 Association between ADC change and pCR in image quality sub-cohorts.

Cohort	pCR		Non-pCR		Diff of ADC change (95% CI) (%)	AUC (95% CI)	p
	N	ADC change (median [IQR]) (%)	N	ADC change (median [IQR]) (%)			
Inadequate sub1	12	17.4 (2.002, 29.7)	43	10.7 (2.6, 20.1)	3.7 (-7.5, 19.4)	0.57* (0.35, 0.80)	0.45
Inadequate sub2	12	30.1 (17.6, 35.1)	25	9.5 (2.2, 21.7)	16.4 (5.9, 26.1)	0.78* (0.60, 0.96)	0.0055
Consensus Adequate	40	16.2 (4.1, 26.2)	93	9.4 (2.6, 17.0)	5.0 (-0.3, 10.6)	0.60 (0.49, 0.71)	0.065

ADC: apparent diffusion coefficient. pCR: pathologic complete response. N: number of patients. Inadequate sub1: patients with inadequate diffusion-weighted image quality (poor quality) at both T0 and T1 ranked by both readers. Inadequate sub2: patients with inadequate diffusion-weighted image quality (poor quality) at either T0 or T1, not both, ranked by both readers. Consensus Adequate: patients with adequate diffusion-weighted image quality at both T0 and T1, ranked by both readers. N: number of patients. CI: confidence interval. Diff: median differences between pCR and non-pCR groups, with estimated 95% confidence intervals. IQR: inter-quartile range. *p=0.16 for the difference.

Table S6 Subtype distributions across quality groups

Cohort	HR+/HER2-	HER2+	TNBC	p-value
Adequate (n=202)	105 (52%)	4 (2%)	93 (46%)	0.034
Inadequate (n=92)	46 (50%)	8 (9%)	38 (41%)	

p-values were calculated by Fisher's exact test.

Figure S1 Example images with different quality rankings. Each example contains four representative images: b0, b800, subtracted DCE, and pre-contrast DCE. The subtracted DCE is a reference for locating the tumor and the pre-contrast DCE is a reference for fat suppression. The quality rankings by two readers are shown at the bottom of each example. (a) shows an example of high-quality DWI which is free from insufficient fat suppression and artifacts, and the signal-to-noise ratio is sufficient. (b) shows an example of poor fat suppression. (c) shows insufficient fat suppression which is more severe in b800 compared to b0. (d) shows an example of a severe ghosting artifact that superimposes the tumor area. (e) shows the distortion in DWI which is considered as an issue in the artifact category. (f) shows an example of poor signal-to-noise ratio (SNR), especially in the b800 DWI. (g) shows the DW images with low SNR and ranked 2 by both readers.

a. High quality

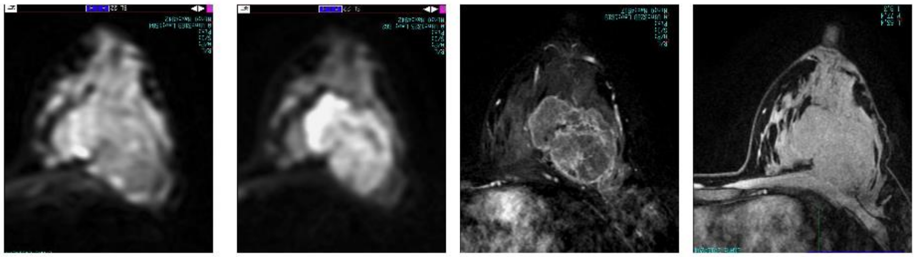

DWI b=0 | DWI b=800 | DCE subtracted | DCE pre-contrast

Quality ranking

	Fat	Artifact	Signal-to-noise ratio
Reader 1	3	3	3
Reader 2	3	3	3

b. Fat suppression (ranked 1)

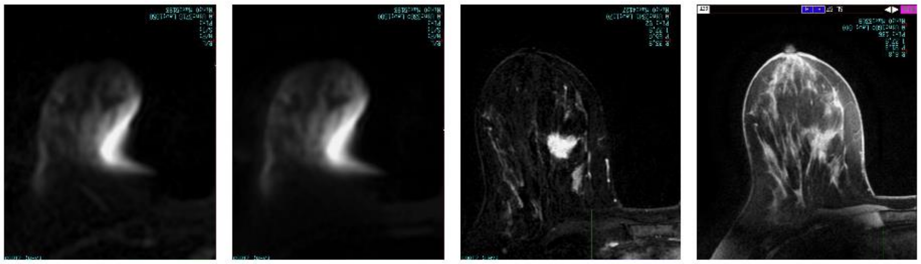

DWI b=0 | DWI b=800 | DCE subtracted | DCE pre-contrast

Quality ranking

	Fat	Artifact	Signal-to-noise ratio
Reader 1	1	1	2
Reader 2	1	1	1

c. Fat suppression (ranked 2)

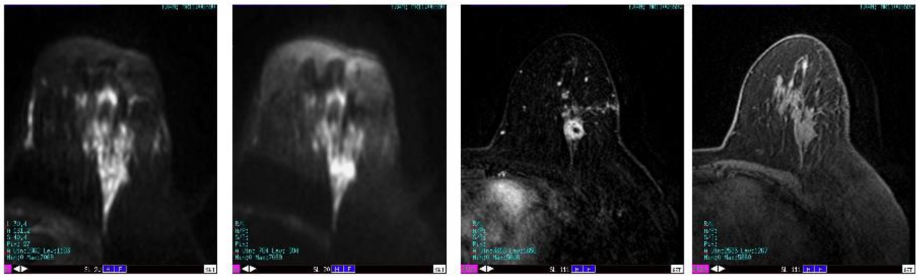

DWI b=0 | DWI b=800 | DCE subtracted | DCE pre-contrast

Quality ranking

	Fat	Artifact	Signal-to-noise ratio
Reader 1	2	2	3
Reader 2	2	1	2

d. Artifact (ranked 1)

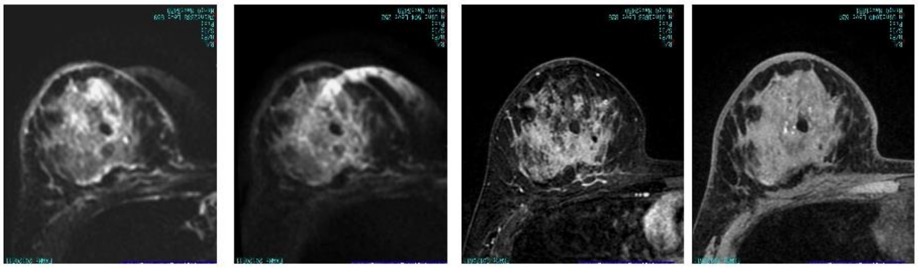

DWI b=0 | DWI b=800 | DCE subtracted | DCE pre-contrast

Quality ranking

	Fat	Artifact	Signal-to-noise ratio
Reader 1	3	1	3
Reader 2	3	1	2

e. Artifact (ranked 2)

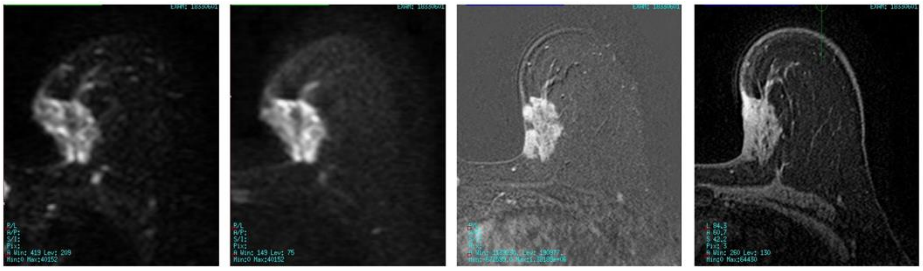

DWI b=0 | DWI b=800 | DCE subtracted | DCE pre-contrast

Quality ranking

	Fat	Artifact	Signal-to-noise ratio
Reader 1	3	2	2
Reader 2	2	2	2

f. Signal-to-noise ratio (SNR, ranked 1)

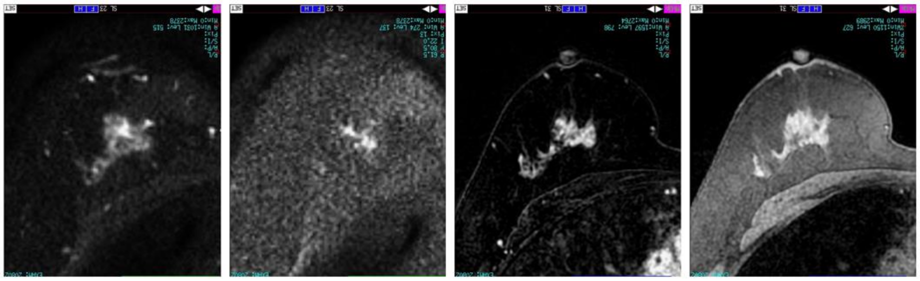

DWI b=0 | DWI b=800 | DCE subtracted | DCE pre-contrast

Quality ranking

	Fat	Artifact	Signal-to-noise ratio
Reader 1	2	1	1
Reader 2	1	1	1

g. Signal-to-noise ratio (SNR, ranked 2)

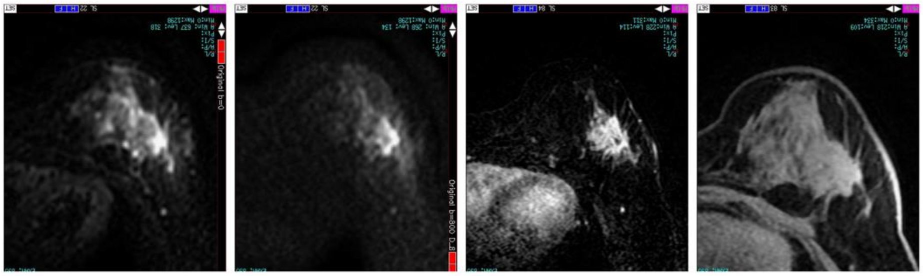

DWI b=0 | DWI b=800 | DCE subtracted | DCE pre-contrast

Quality ranking

	Fat	Artifact	Signal-to-noise ratio
Reader 1	2	3	2
Reader 2	2	2	2

Figure S2 inter-reader agreement of DW image quality among 3 readers
To further understand the inter-reader agreement, one extra reader was recruited to rank the diffusion-weighted (DW) image quality of 26 patients (28 exams in total). This extra reader (Reader 3) was a Radiologist specializing in breast imaging.
First, we verified the agreement between Readers #1 and #2. Results showed consistent agreement with the main analysis with a slightly lower signal-to-noise ratio (SNR). This result is consistent with the main analysis. See the details below.

Reader 1 vs Reader 2

A. Fat

Reader 1 \ Reader 2	1	2	3
1	4	0	0
2	8	10	2
3	0	3	1

B. Artifact

Reader 1 \ Reader 2	1	2	3
1	5	1	0
2	2	12	2
3	0	5	1

C. Signal-to-noise ratio

Reader 1 \ Reader 2	1	2	3
1	2	1	0
2	6	14	2
3	0	3	0

D. Cohen's Kappa

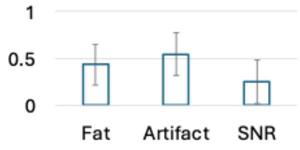

Kappa
Fat: 0.43 (95% CI: 0.22, 0.65)
Artifact: 0.55 (95% CI: 0.32, 0.77)
SNR: 0.25 (95% CI: 0.02, 0.48)

Next, we evaluated the agreement between Readers #1 and #3 (the Radiologist). Results showed similar agreements as between Readers #1 and #2 regarding fat suppression and artifact. However, the agreement on SNR was exceptionally low. See the details below.

Reader 1 vs Reader 3

A. Fat

Reader 1 \ Reader 3	1	2	3
1	2	2	0
2	1	13	6
3	0	2	2

B. Artifact

Reader 1 \ Reader 3	1	2	3
1	5	1	0
2	2	11	3
3	1	3	2

C. Signal-to-noise ratio

Reader 1 \ Reader 3	1	2	3
1	2	1	0
2	4	9	9
3	1	2	0

D. Cohen's Kappa

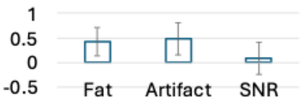

Kappa
Fat: 0.42 (95% CI: 0.14, 0.71)
Artifact: 0.48 (95% CI: 0.16, 0.80)
SNR: 0.091 (95% CI: -0.23, 0.41)

Lastly, we evaluated the agreement between Readers #2 and #3 (the Radiologist). These two readers had very low agreement on fat suppression, lower agreement than Readers #1 and #2, and Readers #1 and #3, higher agreement on SNR than the other two pairs. See the details below.

Reader 2 vs Reader 3

A. Fat

Reader 2 \ Reader 3	1	2	3
1	2	6	4
2	1	9	3
3	0	2	1

B. Artifact

Reader 2 \ Reader 3	1	2	3
1	4	2	1
2	4	11	3
3	0	2	1

C. Signal-to-noise ratio

Reader 2 \ Reader 3	1	2	3
1	5	1	2
2	2	10	6
3	0	1	1

D. Cohen's Kappa

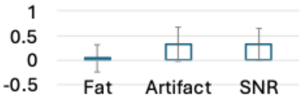

Kappa
Fat: 0.042 (95% CI: -0.24, 0.32)
Artifact: 0.32 (95% CI: -0.024, 0.67)
SNR: 0.33 (95% CI: 0.0009, 0.66)

When the three-level, three-category ranking was consolidated into a two-level ranking, each exam was categorized as having either adequate or inadequate image quality. The percent agreement between Readers #1 and #2 was 71%, consistent with the main

analysis. The percent agreement between Readers #1 and #3 was 82%, the highest among the three pairs. The percent agreement between Readers #2 and #3 was 61%, the lowest among the three pairs.

Figure S3 Inter-reader agreement for DW image quality by field strength. a. Each reader ranked 452 diffusion-weighted MRI exams acquired by 1.5T MRI scanners at pretreatment and early treatment time points for 226 patients, with two exams per patient. b. Each reader ranked 136 diffusion-weighted MRI exams acquired by 3T MRI scanners at pretreatment and early treatment time points for 68 patients, with two exams per patient. A score of 1, 2, or 3 was given to rank the image quality of fat suppression (A), artifact (B), and signal-to-noise (SNR) (C), “1” for low, “2” for medium, and “3” for high image quality. The numbers on the main diagonal of the matrix counted the number of agreements, and the off-diagonal numbers counted the number of disagreements. The difference in Cohen’s Kappa for 1.5T and 3T was not statistically significant for fat suppression (-0.06 [95%CI: -0.17, 0.05], p = 0.30), artifact (-0.09 [95%CI: -0.20, 0.013], p = 0.084), and SNR (-0.12 [95%CI: -0.25, 0.005], p = 0.0599).

A. Fat

Reader 1 \ Reader 2	1	2	3
1	17	5	0
2	85	124	63
3	0	70	88

B. Artifact

Reader 1 \ Reader 2	1	2	3
1	76	28	0
2	47	96	110
3	0	46	49

C. Signal-to-noise ratio

Reader 1 \ Reader 2	1	2	3
1	25	36	0
2	46	165	59
3	0	83	38

Kappa
Fat: 0.45 (95% CI: 0.39, 0.50)
Artifact: 0.52 (95% CI: 0.47, 0.58)
SNR: 0.35 (95% CI: 0.28, 0.41)

a. Field strength 1.5T n=452 exams

A. Fat

Reader 1 \ Reader 2	1	2	3
1	19	6	0
2	29	34	15
3	0	18	15

B. Artifact

Reader 1 \ Reader 2	1	2	3
1	24	5	0
2	17	35	26
3	0	8	21

C. Signal-to-noise ratio

Reader 1 \ Reader 2	1	2	3
1	16	11	0
2	15	42	20
3	0	19	13

Kappa
Fat: 0.51 (95% CI: 0.41, 0.61)
Artifact: 0.62 (95% CI: 0.52, 0.71)
SNR: 0.47 (95% CI: 0.36, 0.58)

b. Field strength 3T n=136 exams

Figure S4 Inter-reader agreement for DW image quality by age group. A. Each reader ranked 300 diffusion-weighted MRI exams acquired for 150 patients < 50 years old at pretreatment and early treatment time points, with two exams per patient. B. Each reader ranked 282 diffusion-weighted MRI exams acquired for 141 patients >= 50 years old at pretreatment and early treatment time points, with two exams per patient. A score of 1, 2, or 3 was given to rank the image quality of fat suppression (A), artifact (B), and signal-to-noise (SNR) (C), “1” for low, “2” for medium, and “3” for high image quality. The numbers on the main diagonal of the matrix counted the number of agreements, and the off-diagonal numbers counted the number of disagreements.

A. Fat

Reader 1 \ Reader 2	1	2	3
1	9	3	0
2	53	82	45
3	0	45	63

B. Artifact

Reader 1 \ Reader 2	1	2	3
1	44	14	0
2	32	66	80
3	0	23	41

C. Signal-to-noise ratio

Reader 1 \ Reader 2	1	2	3
1	16	20	0
2	33	89	46
3	0	62	34

Kappa
Fat: 0.44 (95% CI: 0.37, 0.51)
Artifact: 0.53 (95% CI: 0.46, 0.60)
SNR: 0.35 (95% CI: 0.27, 0.43)

A. Patients < 50 years old n=300 exams

A. Fat

Reader 1 \ Reader 2	1	2	3
1	26	7	0
2	61	75	32
3	0	42	39

B. Artifact

Reader 1 \ Reader 2	1	2	3
1	55	19	0
2	32	64	54
3	0	29	29

C. Signal-to-noise ratio

Reader 1 \ Reader 2	1	2	3
1	24	27	0
2	27	114	33
3	0	40	17

Kappa
Fat: 0.49 (95% CI: 0.42, 0.56)
Artifact: 0.56 (95% CI: 0.49, 0.62)
SNR: 0.39 (95% CI: 0.31, 0.48)

B. Patients >= 50 years old n=282 exams

Figure S5 Inter-reader agreement for DW image quality by chemotherapy regimen. A. Each reader ranked 292 diffusion-weighted MRI exams acquired for 146 patients treated with standard chemotherapy at pretreatment and early treatment time points, with two exams per patient. B. Each reader ranked 296 diffusion-weighted MRI exams acquired for 148 patients treated with an experimental regimen at pretreatment and early treatment time points, with two exams per patient. A score of 1, 2, or 3 was given to rank the image quality of fat suppression (A), artifact (B), and signal-to-noise (SNR) (C), "1" for low, "2" for medium, and "3" for high image quality. The numbers on the main diagonal of the matrix counted the number of agreements, and the off-diagonal numbers counted the number of disagreements.

A. Fat

Reader 1 \ Reader 2	1	2	3
1	17	4	0
2	60	77	31
3	0	50	53

B. Artifact

Reader 1 \ Reader 2	1	2	3
1	57	23	0
2	20	58	69
3	0	31	34

C. Signal-to-noise ratio

Reader 1 \ Reader 2	1	2	3
1	23	27	0
2	34	105	18
3	0	66	19

Kappa
Fat: 0.48 (95% CI: 0.42, 0.55)
Artifact: 0.56 (95% CI: 0.50, 0.63)
SNR: 0.38 (95% CI: 0.30, 0.46)

A. Standard chemotherapy n = 292 exams

A. Fat

Reader 1 \ Reader 2	1	2	3
1	19	7	0
2	54	81	47
3	0	38	50

B. Artifact

Reader 1 \ Reader 2	1	2	3
1	43	10	0
2	44	73	67
3	0	23	36

C. Signal-to-noise ratio

Reader 1 \ Reader 2	1	2	3
1	18	20	0
2	27	102	61
3	0	36	32

Kappa
Fat: 0.47 (95% CI: 0.39, 0.54)
Artifact: 0.52 (95% CI: 0.45, 0.59)
SNR: 0.39 (95% CI: 0.30, 0.47)

B. Experimental regimen n = 296 exams

Figure S6 Boxplots of ADC change versus pathologic complete responses in the quality inadequate sub-cohorts. P-values were 0.45 and 0.0055 for the difference between pCRs and non-pCRs, respectively. The statistically significant levels were labeled at the top ("ns" represents not significant).

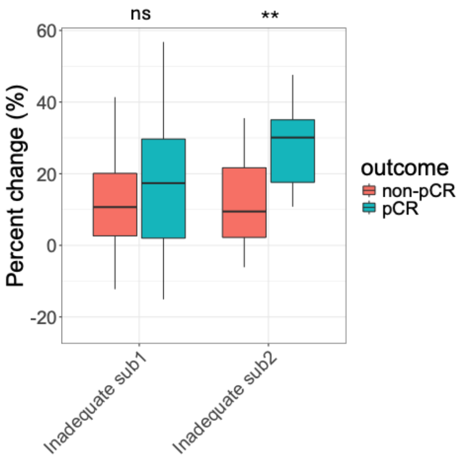

Figure S7 Multivariate analysis of interaction between ADC change and breast cancer molecular subtype (HR+/HER2-, HER2+, TNBC)

Likelihood ratio test
R code

```
model_no_interaction <- glm(pCR ~ ADC.pct + subtype, family = binomial(link = "logit"), data=dat1)
model_with_interaction <- glm(pCR ~ ADC.pct * subtype, family = binomial(link = "logit"), data=dat1)
#likelihood ratio test
anova(model_no_interaction, model_with_interaction, test = "Chisq"
```

In the full analysis cohort n=294 p=0.15

```
Model 1: pCR ~ ADC.pct + subtype
Model 2: pCR ~ ADC.pct * subtype
  Resid. Df Resid. Dev Df Deviance Pr(>Chi)
1       290     332.33
2       288     328.56  2   3.7692   0.1519
```

In the quality adequate cohort n=202 p=0.95

```
Model 1: pCR ~ ADC.pct + subtype
Model 2: pCR ~ ADC.pct * subtype
  Resid. Df Resid. Dev Df Deviance Pr(>Chi)
1       198     239.08
2       196     238.99  2 0.095608   0.9533
```

In the quality inadequate cohort n=92 p=0.01069

```
  Resid. Df Resid. Dev Df Deviance Pr(>Chi)
1        88     89.576
2        86     80.499  2   9.0772  0.01069 *
---
Signif. codes:  0 ‘***’ 0.001 ‘**’ 0.01 ‘*’ 0.05 ‘.’ 0.1 ‘ ’ 1
```

In the quality inadequate sub1 cohort n=55 p=0.041

```
Model 1: pCR ~ ADC.pct + subtype
Model 2: pCR ~ ADC.pct * subtype
  Resid. Df Resid. Dev Df Deviance Pr(>Chi)
1        51     50.510
2        49     44.128  2   6.3819  0.04113 *
---
Signif. codes:  0 ‘***’ 0.001 ‘**’ 0.01 ‘*’ 0.05 ‘.’ 0.1 ‘ ’ 1
```

In the quality inadequate sub2 cohort n=37 p=0.17

```
Analysis of Deviance Table

Model 1: pCR ~ ADC.pct + subtype
Model 2: pCR ~ ADC.pct * subtype
  Resid. Df Resid. Dev Df Deviance Pr(>Chi)
1        33     37.287
2        31     33.768  2   3.5185   0.1722
```

It appears that the interaction between ADC change and breast cancer subtype was statistically significant in the quality inadequate group. We subsequently did subtype-specific analysis.

Subtype-specific estimates showed that each 1-unit increase in ADC.pct was associated with a 6% increase in the odds of pCR in TNBC (OR 1.06, 95% CI 1.00–1.12, p = 0.034), no clear association in HR+/HER2− (OR 0.96, 95% CI 0.90–1.03, p = 0.29), and a larger but imprecisely estimated increase in HER2+ (OR 1.29, 95% CI 0.87–1.91, p = 0.20).

Pairwise comparisons of subtype-specific effects were not statistically significant. The relative effect of ADC.pct was 34% higher in HER2+ compared with HR+/HER2− (OR 1.34, 95% CI 0.90–1.99, p = 0.32) and 22% higher compared with TNBC (OR 1.22, 95% CI 0.82–1.81, p = 0.59). There was weak evidence that the effect of ADC.pct was lower in HR+/HER2− compared with TNBC (OR 0.91, 95% CI 0.84–0.99, p = 0.087).

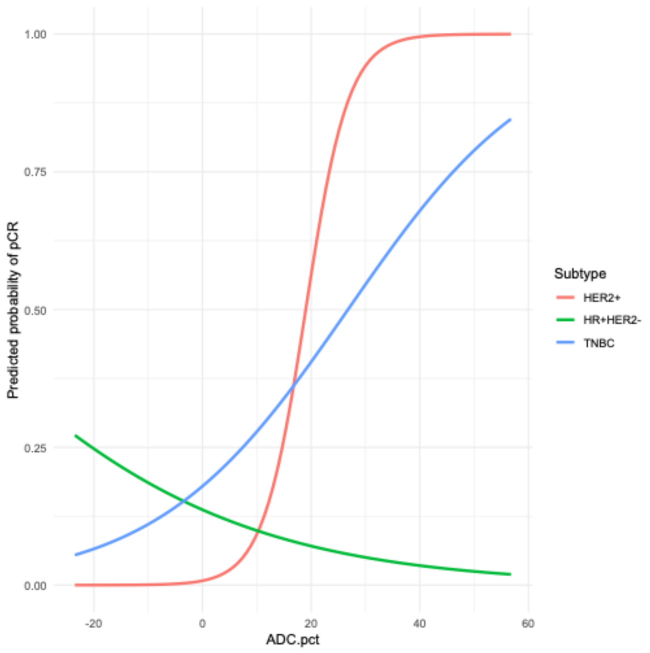

We plotted predicted probability over ADC.pct by subtype (see above). The plots suggested that these relationships may be nonlinear, particularly within the HR+/HER2- subgroup, and a spline-based sensitivity analysis demonstrated improved fit compared with the linear interaction model (p = 0.0045).

Overall, these findings suggest modest heterogeneity in the ADC.pct–pCR association across subtypes, with no individual pairwise contrast reaching statistical significance despite evidence of a global interaction.
